# Eye-tracking-based assessment of cognitive function in low-resource settings

**DOI:** 10.1136/archdischild-2016-310525

**Published:** 2016-08-22

**Authors:** Linda Forssman, Per Ashorn, Ulla Ashorn, Kenneth Maleta, Andrew Matchado, Emma Kortekangas, Jukka M Leppänen

**Affiliations:** 1 Tampere Center for Child Health Research, School of Medicine, University of Tampere, Tampere, Finland; 2 Department of Paediatrics, Tampere University Hospital, Tampere, Finland; 3 School of Public Health and Family Medicine, College of Medicine, University of Malawi, Blantyre, Malawi

**Keywords:** infant, Neurodevelopment, cognition

## Abstract

**Background:**

Early development of neurocognitive functions in infants can be compromised by poverty, malnutrition and lack of adequate stimulation. Optimal management of neurodevelopmental problems in infants requires assessment tools that can be used early in life, and are objective and applicable across economic, cultural and educational settings.

**Objective and design:**

The present study examined the feasibility of infrared eye tracking as a novel and highly automated technique for assessing visual-orienting and sequence-learning abilities as well as attention to facial expressions in young (9-month-old) infants. Techniques piloted in a high-resource laboratory setting in Finland (N=39) were subsequently field-tested in a community health centre in rural Malawi (N=40).

**Results:**

Parents' perception of the acceptability of the method (Finland 95%, Malawi 92%) and percentages of infants completing the whole eye-tracking test (Finland 95%, Malawi 90%) were high, and percentages of valid test trials (Finland 69–85%, Malawi 68–73%) satisfactory at both sites. Test completion rates were slightly higher for eye tracking (90%) than traditional observational tests (87%) in Malawi. The predicted response pattern indicative of specific cognitive function was replicated in Malawi, but Malawian infants exhibited lower response rates and slower processing speed across tasks.

**Conclusions:**

High test completion rates and the replication of the predicted test patterns in a novel environment in Malawi support the feasibility of eye tracking as a technique for assessing infant development in low-resource setting. Further research is needed to the test–retest stability and predictive validity of the eye-tracking scores in low-income settings.

**What is already known on this topic?**

- Optimal management of neurodevelopment problems in infants requires assessment tools that are applicable across cultural, educational, and economic settings.
- Eye-tracking applications have been developed for the assessment of visual and cognitive functions in high-resource laboratories.

**What this study adds?**

- This study demonstrates the feasibility of eye tracking in rural Malawi, supporting its use as a technique for assessing infant cognitive development in low-resource settings.

## Introduction

Human brain development is highly dependent on access to ‘optimal’ environment during the first years of life^1^ and can be compromised in children born in settings with scarce access to nutrition and cognitive stimulation.^1–5^ To understand the prevalence and burden of children's neurodevelopmental problems in such settings, it is critical to develop assessment techniques that are applicable across economic, cultural and educational contexts, and allow for early identification of children at risk for long-term cognitive deficits.

Existing techniques for assessing early cognitive development in infants^6^ are limited by the requirement for manual test administration and subjective judgements of infant behaviour. As such, the tests are time consuming, error prone and difficult to standardise for widescale use. The tests have also been criticised for a lack of sensitivity to specific neurocognitive processes.^7^ Because of these limitations, there is interest in the potential utility of alternative technologies, such as automated tracking of infants' eye movements^8–10^ in assessing early cognitive development.

Automated eye-tracking-based tests have been developed for assessing infants' visual acuity,^11^ visuospatial orienting (gaze shifts to novel stimuli)^12^ ^13^ and attention to salient social cues, such as faces.^14^ These cognitive processes provide a ‘building block’^7^ for the development of more advanced cognitive and social skills and, as such, can provide useful markers of early cognitive development in infants. For example, tests assessing the speed of visuospatial orienting at 3.5 or 7 months of age predict cognitive performance later in childhood, as assessed by standardised IQ tests at age 4 or tests of executive function at age 11.^15^ ^16^ Similarly, tests assessing infants' attentional bias for faces at 7 months predict socioemotional development at the age of 14 months.^17^

Eye-tracking technologies have been extensively used in Europe, North America and Japan, but there is a paucity of studies using this method in low-income countries. Cultural differences, such as unfamiliarity with the concept of infant cognitive testing, lack of experience of screen-based stimuli and test settings, could interfere with the method being successfully implemented in low-income settings.

We examined the feasibility of eye tracking as a novel technique for assessing infants' cognitive function in low-resource settings. We created a battery of tests that have been previously used in high-resource settings, piloted the test battery in a high-resource laboratory in Finland and subsequently tested it in a rural low-income environment in Malawi. As a direct test of feasibility, we compared the two test sites for (1) parents' perception of the acceptability of the method, (2) test completion rates and (3) patterns of infants' test responses. Similar comparisons were conducted for traditional observational assessments of infant cognition. For the eye-tracking method to be defined as feasible, we expected (1) that most parents accepted the method in Finland and Malawi, and test completion rates in Finland and Malawi were comparable and within the range reported in previous studies (82–100% for test completion rates and 72–86% for percentage of valid trials;^12^ ^13^ ^18^ (2) a replication of the predicted pattern of test responses in Malawian infants, (ie, active visual search of a target and sensitivity to visual interference, anticipatory saccades and perceptual learning, and heightened attention to facial expressions) and (3) similarity of the feasibility indices for eye-tracking and observational tests.

## Method

### Participants

Based on previous studies,^19–21^ a sample size of 30–40 infants per group was expected to be sufficient for testing feasibility and detecting group differences. To be included in the study, the child's biological mother had to come to the study site and speak the local language fluently. Exclusion criteria included preterm birth (<37 gestational weeks), low birth weight (<2500 g) and known visual impairment, neurological disorder or congenital malformation, based on parent report. The Finnish sample was recruited by sending invitations to families with a <9-month-old infant in Tampere city area (population 222 500). Families indicating interest to participate were contacted by phone and screened for inclusion and exclusion criteria. The Malawian sample was recruited by providing verbal information about the study to village chiefs and community advisers in the Lungwena health centre area (approximate population 30 000). If a mother indicated interest in participating, her child was screened for appropriate age and an appointment to the health centre was made for further screening and determination of eligibility for enrolment in the study.

Each study visit took 1–1.5 hours to complete. Finnish participants received a T-shirt worth ∼€5 for participating and Malawian participants a compensation for travel cost and 1 kg of rice (∼€0.75). The study protocol was approved by the ethics committees of the College of Medicine, Malawi, Pirkanmaa Hospital District and University of Tampere, Finland. Written informed consent was obtained from the participants' biological mother.

### Assessment of infants' cognitive and social function

#### Eye tracking

**Figure 1:**
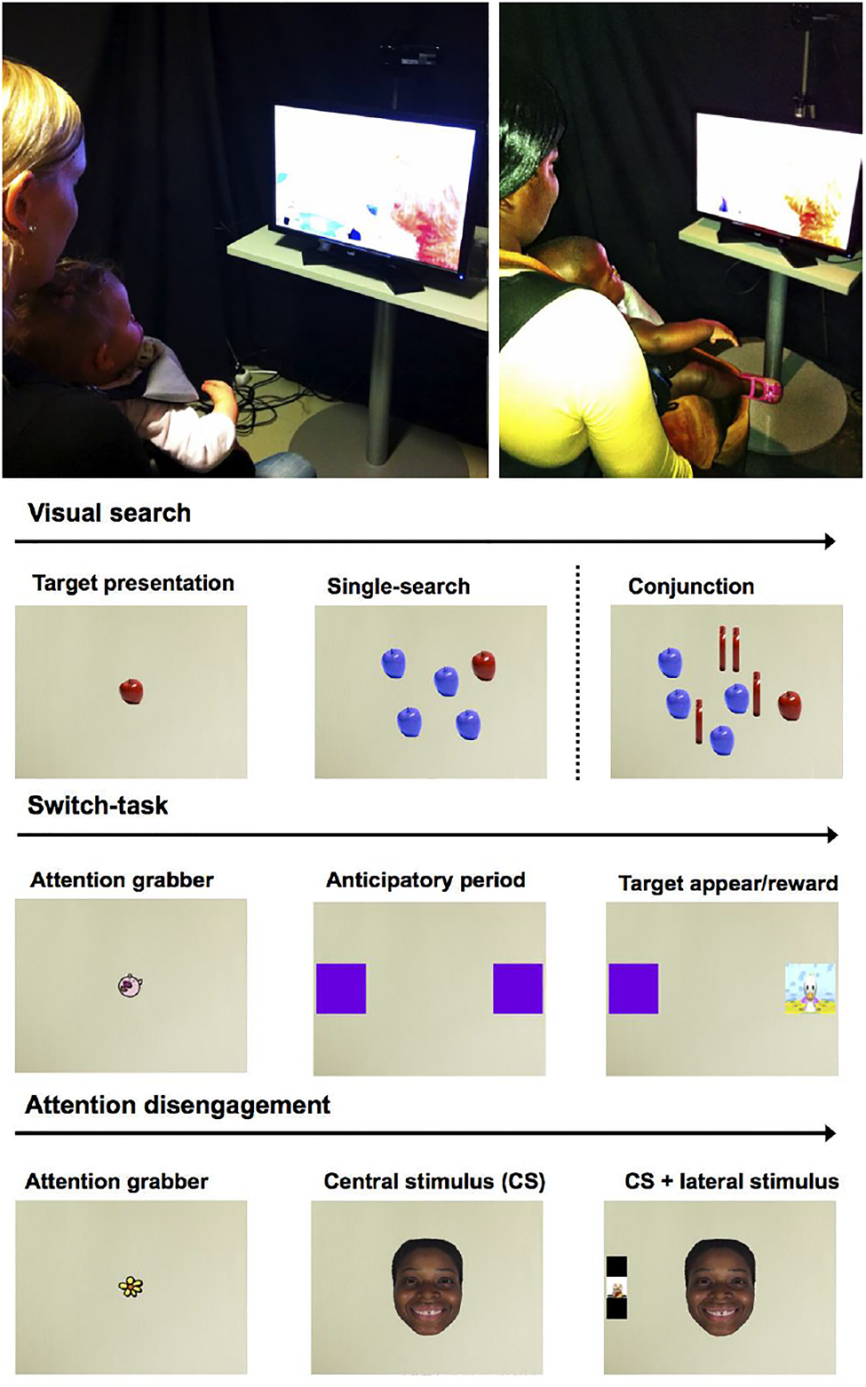
An illustration of eye-tracking sessions in Finland (top left) and Malawi (top right), and paradigms designed to test infants’ visual orientation abilities (visual search), sequence-learning abilities (switch task) and attention to facial expressions of emotion (disengagement).

Eye-tracking assessment took place in a quiet and dimly lit room (figure 1). The infants were seated on their mother's lap at an ∼60 cm viewing distance in front of a 22-inch monitor and a Tobii X2-60 eye tracker (Tobii Technology, Stockholm, Sweden). Three tests of early cognitive function were administered: a Visual Search task^19^—assessing the ability to search for a target (a red apple) when presented alone (one-object condition), or among distractors of one kind (distractor condition), or two kinds (conjunction condition); a Switch-Task^20^—assessing the ability to learn to anticipate the side where a target would appear in the preswitch and postswitch phases; and a Disengagement task^22^—assessing the disengagement of attention from a non-face pattern or a happy or fearful face to a salient lateral stimulus.

Eye-tracking data were analysed using automated MATLAB scripts (MathWorks, Natick, Massachusetts, USA) and similar criteria as those used in prior studies.^13^ For each of the three tasks, the proportion of test trials with a gaze shift to the target stimulus in a prespecified time window out of all scorable trials for the task were coded and served as a dependent variable in the analyses. A measure of the infants' overall processing speed was created by averaging gaze shift latencies across the three tasks.

#### Structured observation

Traditional structured observation tests were administered to assess infants' ability to respond to and initiate non-verbal social communication.^23^ Video records of infants' behaviour during the test were analysed for the proportion of correct gaze following and alternating responses (see online supplementary information for details).

10.1136/archdischild-2016-310525.supp1 Supplementary data

### Mother's perception of the acceptability of the eye tracking

Mothers were asked (1) whether they had enjoyed participating with their child in the assessment and (2) whether they would recommend a friend to participate in the assessment with their child. The responses were marked as ‘yes’ or ‘no’.

### Socio-demographics and anthropometric assessment

Descriptive data were collected by interviewing the mothers using a socio-demographic questionnaire, including questions about maternal age, parental education level and family structure. Infants' length was measured to the nearest 1 mm by using a length board (Harpenden Infantometer, Holtain Limited, Crosswell, UK) and infant weight by using an electronic infant weighing scale (SECA 735) with reading increments of 10 g. Mid upper arm circumference (MUAC) and head circumference were measured to the nearest 1 mm with non-stretchable plastic insertion tapes. Anthropometric measurements were done in triplicate by trained personnel, and the mean of the triplicate measurements was used to calculate age-standardised and sex-standardised anthropometric indices (weight-for-age, length-for-age, weight-for-length, head circumference-for-age and MUAC-for-age Z-scores) using the WHO standards.^24^

### Statistical analysis

The samples were compared by using Pearson's χ^2^ tests for dichotomous variables (ie, parental acceptance measures, test completion rates and valid trials), t-tests for normally distributed continuous variables and non-parametric Mann-Whitney U tests for non-normal continuous variables.

## Results

A total of 39 Finnish and 40 Malawian infants were enrolled for the study in May/June 2014 (Finland) and September 2014 (Malawi). Three of the Malawian infants did not participate in eye tracking because their mothers opted out (n=2) or because the assessments could not be conducted (n=1; fussy infant). No data were obtained from these participants, but parental acceptance rates were calculated by including the two mothers who opted out, and test completion rates by including the infant who was not assessed due to fussiness (ie, on an intent-to-test basis). All other statistics, including sample descriptives (table 1), percentages of valid test trials (table 2) and test scores (table 3), are reported for participants assessed with eye tracking.

**Table 1:** Description of the sex, age, socio-demographic and anthropometric measures of infants who were assessed with the eye-tracking tests

	Malawian infants (n=37)	Finnish infants (n=39)	p Value*
Sex			
Females, N and percentage	18 (48.6%)	20 (51.3%)	0.818
Age, days	274 (6)†	274 (4)	0.942
Socio-demographics			
Mothers' years of education	3.4 (3.9)	16.1 (2.6)	<0.001
Fathers’ years of education	5.1 (4.4)	15.4 (2.6)	<0.001
Mothers’ age, years	24 (5)	32 (5)	<0.001
No. of children in the household	2.4 (1.2)	1.7 (1.2)	0.030
No. of people in the household	4.8 (1.9)	3.8 (1.2)	0.055
Child anthropometrics			
Weight, kg	7.95 (0.98)	9.09 (1.03)	<0.001
Length, cm	67.0 (2.1)	73.3 (2.5)	<0.001
MUAC, cm	14.5 (1.2)	15.5 (1.2)	0.001
Head circumference, cm	44.0 (1.3)	45.3 (1.2)	<0.001
Weight-for-age	−0.71 (1.01)	0.48 (0.98)	<0.001
Weight-for-length	0.39 (1.10)	0.08 (0.87)	0.178
Length-for-age	−1.76 (0.80)	0.98 (1.07)	<0.001
Head circumference-for-age	−0.55 (0.93)	0.71 (0.84)	<0.001
MUAC-for-age	0.11 (1.04)	0.95 (1.02)	0.001

*Means were compared with t-tests, percentage differences and nominal data were compared with χ^2^.

†Mean (SD) all such values.

MUAC, mid upper arm circumference.

**Table 2:** Test completion rates (number of participants completing the assessments), the overall number and percentage of valid trials, and the median and IQR for the number of valid trials per child for the eye-tracking tests

	Malawi	Finland	p Value*
Eye tracking			
Overall completion rate	34/38 (89.5%)	37/39 (94.9%)	0.377
Visual search			
Completion rate	33/38 (86.8%)	37/39 (94.9%)	0.220
Valid trials	642/876 (73.2%)	820/960 (85.4%)	<0.001
Valid trials per child	18.0 (4.5)	22.0 (5.0)	<0.001
Switch task			
Completion rate	35/38 (92.1%)	39/39 (100.0%)	0.073
Valid trials	807/1157 (69.7%)	992/1261 (78.7%)	<0.001
Valid trials per child	22.0 (7.5)	27.0 (7.0)	0.004
Disengagement			
Completion rate	34/38 (89.5%)	36/39 (92.3%)	0.665
Valid trials	785/1153 (68.1%)	864/1248 (69.2%)	0.320
Valid trials per child	22.0 (9.5)	23.0 (10.0)	0.405
*Structured observation*			
Gaze following			
Completion rate	33/38 (86.8%)	39/39 (100.0%)	0.019
Valid trials	257/264 (97.4%)	310/312 (99.4%)	0.086
Valid trials per child	8.0 (0.0)	8.0 (0.0)	0.076
Alternating gaze			
Completion rate	33/38 (86.8%)	39/39 (100.0%)	0.019
Valid trials	277/297 (93.3%)	342/351 (97.4%)	0.088
Valid trials per child	9.0 (1.0)	9.0 (0.0)	0.012

*Proportions were compared with Pearson's χ^2^ tests and median trials per child with Mann-Whitney U tests.

**Table 3:** Scores on the eye-tracking and structured observation assessments by site

	Malawian infants Mean (95% CI)	Finnish infants Mean (95% CI)	p Value*
Visual search			
One-object (p)†	0.87 (0.81 to 0.93)	0.96 (0.91 to 1.00)	0.001
Distractor (p)	0.39 (0.32 to 0.47)	0.51 (0.43 to 0.59)	0.038
Conjunction (p)	0.33 (0.24 to 0.42)	0.36 (0.29 to 0.42)	0.482
Switch-task			
Preswitch (p)	0.70 (0.62 to 0.79)	0.70 (0.61 to 0.78)	0.99
Postswitch (p)	0.52 (0.46 to 0.61)	0.47 (0.38 to 0.56)	0.30
Disengagement			
Control (p)	0.94 (0.91 to 0.97)	0.94 (0.91 to 0.98)	0.626
Happy (p)	0.43 (0.34 to 0.51)	0.71 (0.61 to 0.80)	<0.001
Fear (p)	0.31 (0.22 to 0.41)	0.64 (0.53 to 0.74)	<0.001
Processing speed			
Reaction time (ms)	450.76 (425.6 to 476.0)	398.46 (372.9 to 424.06)	0.003
Structured observation tasks			
Gaze following (p)	0.43 (0.36 to 0.51)	0.46 (0.39 to 0.53)	0.541
Alternating gaze (p)	0.47 (0.35 to 0.59)	0.47 (0.38 to 0.55)	0.816

*Refers to the significance testing of group differences. All comparisons were conducted by Mann-Whitney U tests except that for the processing speed, which was conducted by t-test.

†Proportion of correct responses.

The Finnish and Malawian infants assessed with eye tracking did not differ in sex ratios or age, but significant group differences were found in socio-demographic and anthropometric variables (table 1).

### Feasibility of the eye-tracking method

In total, 95% (N=37) of the Finnish mothers and 92% (N=36) of the Malawian mothers reported that they enjoyed participating in the eye-tracking assessment with their child, and 100% (N=39) of Finnish and 95% (N=37) of Malawian mothers stated they would recommend a friend to participate with their child. And 95% (N=37) of the Finnish and 89.5% of the Malawian (N=34) infants completed the whole test battery (∼15–25 min of testing). For five infants, the assessment was terminated because the infant became too tired/inattentive (Finnish: n=2; Malawian: n=1), a discontinuation in electricity supply (Malawian: n=1) or the infant reacted negatively to the stimuli in one task (Malawian: n =1). These five infants were still able to provide sufficient number of valid trials to meet the inclusion criteria for most of the eye-tracking tasks.

The completion rates for the eye-tracking tasks were slightly higher in Finland than in Malawi, although none of the differences were significant (p values=0.073–0.377, table 2). Finnish children had a significantly higher proportion of valid trials for the visual search task and switch-task (ps<0.01), whereas there was no significant difference for the disengagement task (p=0.320). For the structured observation, there was a significant difference between the Finnish and Malawian sites in the test completion rates (p=0.035) and a marginal difference in the number of valid trials (p=0.09).

### Comparison of Finnish and Malawian infants

The response patterns on the eye-tracking tasks in Malawi were similar to those observed in prior studies and those in the Finnish sample (table 3 and online supplementary figures S2 and S3): the percentage of successful visual search responses decreased linearly with increasing level of visual distraction (indicating sensitivity to visual interference), the infants demonstrated reliable increase in anticipatory saccades for stimuli presented repeatedly in the same spatial location and updated such anticipatory responses based on changes in location (indicating perceptual learning), and infants disengaged more frequently from the non-face control stimulus than from a happy and fearful face (reflecting enhanced attention to faces as social cues).

Compared with Finnish infants, Malawian infants had lower percentage of successful search responses in two search conditions (ps<0.03, table 3), had slightly lower increase in anticipatory saccades over the course of the experiment (p=0.075) and were less likely to disengage from emotional faces (p<0.001). Processing speed was, on average, 50 ms slower in Malawian infants.

There were no significant differences in performance between the two samples on the observational assessments of non-verbal social communication.

## Discussion

We examined the feasibility of eye tracking in socioeconomically diverse settings in Finland and Malawi. Our results showed high maternal acceptance of the method at both sites. Test completion rates were within the expected range (>82%) at both sites and largely comparable to the corresponding values for traditional observational tests. Compared with Finnish infants, Malawian infants had lower percentage of valid test trials in two out of three eye-tracking tasks. The percentage of valid trials were below the expected range (ie, 72%) for one task in Finland and two tasks in Malawi. The deviations from the expected range were, however, relatively small in magnitude at both sites (<4%) and unlikely to have practically meaningful consequences for the feasibility of eye tracking. Together, these results suggest that there are no barriers for conducting eye tracking in low-income environments.

As an additional indicator of transferability of eye tracking, our results showed similar pattern of responses in infants at the two sites. Similar to Finnish infants, Malawian infants exhibited active visual search, anticipatory saccades and prioritised attention to facial expressions. This result suggests that the cognitive constructs that have been documented in studies in Europe, the USA and Japan^19^ ^20^ ^22^ are found in sub-Saharan African infants and can be effectively assessed by eye-tracking methods.

Compared with Finnish infants, Malawian infants had a lower rate of successful visual search responses, longer attention shift times (processing speed) and a lower rate of gaze disengagements from faces. While these findings could be interpreted as an indication of a cognitive disadvantage^25^ and heightened sensitivity to social signals^22^ in infants in adverse environments, we are reluctant to make a strong case for this interpretation at this point given that a variety of alternative explanations cannot be ruled out (eg, familiarity with electronic displays), and longitudinal data on cognitive outcomes were not available. The differences were found in the eye-tracking-based assessment, but not in the more naturalistic structured observations. Eye tracking may be more sensitive to subtle differences in cognitive performance between the two groups or may be capturing different aspects of cognitive function than the observational tasks, but, again, the possibility that Malawian infants are simply less familiar (and therefore more disadvantaged) in perceiving and responding to stimuli on digital displays compared with more naturalistic settings cannot be ruled out.

Our study did not have adequate power to detect small to medium differences (Cohen's d<0.8) in feasibility indices between the sites. It is unlikely, however, that more subtle differences not detected in this study would create major challenges for the implementation of the test. The current study was also limited to one low-income setting. Whereas we consider it unlikely that the implementation of eye tracking would be markedly different in other low-income settings, such heterogeneity cannot be ruled out. These limitations notwithstanding, the present study provides a demonstration of the feasibility of eye tracking in low-resource setting and motivates further studies to examine individual variations in eye-tracking test scores, their psychometric properties and, ultimately, predictive value in low-income settings.
