# Supplementary material for: Eye-tracking-based assessment of cognitive function in low-resource settings
Source: Arch Dis Child. 2016 Aug 22;102(4):301–2. doi: 10.1136/archdischild-2016-310525 (PMC5466915; doi:10.1136/archdischild-2016-310525)
Supplement: Supplementary data [file archdischild-2016-310525supp001.pdf]

Supplemental Information

Eye Tracking Based Assessment of Cognitive Development in Low-Resource Settings

Linda Forssman[1], Per Ashorn[1,2], Ulla Ashorn[1], Kenneth Maleta[3], Andrew Matchado[3], Emma Kortekagas[1] & Jukka M. Leppänen[1]

[1]Tampere Center for Child Health Research, School of Medicine, University of Tampere, Finland; [2]Department of Paediatrics, Tampere University Hospital, Finland; [2]College of Medicine, University of Malawi, Malawi

Corresponding author: Jukka M. Leppänen, Tampere Center for Child Health Research, School of Medicine, University of Tampere, FIN-33014 Tampere, Finland, E-mail: jukka.leppanen@uta.fi, Tel. +358 40 190 1358, Fax: +358 3 213 4473

Eye Tracking Set-Up

The eye tracking assessment took place in a quiet and dimly lit room at the Finnish and Malawian sites. The area where the participants were placed in front of the eye tracker was surrounded by black fabric to create a circumscribed (2 x 2 m) space and to standardize the testing environments for the Finnish and Malawian participants, particularly in terms of luminance. During the testing the only light source came from the monitor and a spotlight (E14 LED light bulb) that was placed slightly behind the participant about 2 meters from the floor and directed down. The eye tracking testing package was written on MATLAB technical computing software (Mathworks, Natick, MA), running on MacBook Pro OS X (Apple Inc.) and interfacing via Psychtoolbox and Tobii SDK plug-in with the eye-tracker and a 22-inch monitor (Dell Inc.). The study utilized a portable Tobii X2-60 eye tracker (Tobii Technology, Stockholm), which tracks the participant's point of gaze at 60 Hz with a spatial accuracy of 0.4° as reported by the manufacturer. All testing equipment (e.g., computer, eye tracker, monitor, speakers, fabric, spotlight) used at the Finnish site was transported to Malawi for testing of the Malawian infants.

Eye Tracking Test Battery

The eye tracking test battery was presented in two sessions and took between 15 to 20 minutes to administer, including breaks for the infant during the testing. In both eye tracking sessions, the same tasks were presented in the same order for each participant. The structured observation was administered between the two eye tracking sessions. The purpose of this design was to allow for a break from the eye tracking assessment, while still continuing with the testing, as infants are limited in their ability to sit still and stay focused on one task.

The eye tracking test battery consisted of three tasks: a visual search task, a switch-task, and an attention disengagement task. In addition, we created an aggregated measure of processing speed based on RTs from the three eye tracking task. Each testing session started with presenting either the attention disengagement task or the switch-task, and this presentation order was balanced in an odd/even number fashion based on the participant number. The visual search task was always presented between the other two tasks, as it was more complex, involving more than the infant to shift his/her gaze vertically to the left and right of the center to find a target.

Before the start of each eye tracking session, an eye tracking calibration procedure was performed. This procedure is critical for obtaining valid and reliable gaze tracking of an individual. During the calibration, five small cartoon figures with accompanying sounds appeared, one at a time, in each corner and in the center of the screen. Any unsuccessful calibration was recalibrated at least two times to reach satisfactory calibration.

The eye tracking test battery was designed to be engaging for infants by using infant-friendly stimuli and gaze-contingent features. Besides making the tasks more engaging, the gaze-contingent features also allowed for a more automatic presentation of the tasks (e.g., a trial commenced after the infant fixated an attention-grabber) and also had the function of rewarding correct performance during the tasks (e.g., in the visual search task the target spun and made sound after a "gaze hit"), which is a particularly applicable feature when testing pre-verbal infants. Further, every trial was preceded by the presentation of gaze-contingent attention-grabber. This was done to attract the infant's attention to the center of the screen and to make sure that the infant's gaze was tracked from the start of each trial.

During the testing, the experimenter sat behind a curtain out of the infant's view. From that position, s/he monitored the infant's behavior through a video camera and the infant's gaze tracked in real time on a computer. If the infant became fidgety or inattentive during the assessment short breaks were taken. If the infant's tracked gaze started to look flickery (i.e., lots of gaps in tracking) the infant's position was adjusted or the testing was paused and the infant's eyes were recalibrated.

Visual Search Task

The infant's ability to search for a target with and without distractors was assessed with a visual search task (modeled on Kaldy et al., 2011). Before the start of each trial, the infants were presented with a familiarization trial where the target, a red apple (5° visual angle), was shown on the center of the screen accompanied with a "oh" sound. Similar to Kaldy et al.'s study (2011), we used a maximum presentation time of 4 s. In contrast to their study, the length of the presentation was gaze-contingent, but with a minimum presentation time of 2 s to ensure that the infant was given sufficient time to view the stimulus. If the infant fixated the target, a search trial commenced after a short delay (500 ms).

The search trials were presented in two blocks (eye tracking session 1 and 2) and in each block they consisted of 4 one-object trials (one target and no distractors), 4 distractor condition trials (2 trials: one target and 4 distractors; 2 trials: one target and 8 distractors), and 4 conjunction trials (2 trials: one target and 4 distractors; 2 trials: one target and 8 distractors). In the distractor condition trials the red apple target was presented with distractors consisting of either blue apples (same shape, but different color; 5° visual angle) or red elongated rectangle sliced apples (same color, but different shape). In the conjunction trials, half of the distractor objects were blue

apples and the other half red sliced apples. If the participant fixated the target within 4 s from the start of the trial, a reward sound (children voices cheering "yeah") was played while the target spun. The same audiovisual effect was presented to the participant if s/he didn't find the target within 4 s to make the task more engaging and to draw attention to the target.

A trial was considered valid if it met the pre-processing criteria for the analysis period (i.e., from 0-1000 ms from the onset of the anticipatory period; see Processing of the eye tracking data). Of the valid data we calculated proportion of successful searches between 100 ms to 1000 ms from the start of the search trial in the single-search one-object trials, single-search multiple-object trials, and in the conjunction trials. A 1000 ms cut-off for missed/successful searches was used to avoid ceiling effects. Data were excluded from infants who failed to provide at least 3 valid trials/condition (single-search one-object, single-search multiple-object, and conjunction; Finland: n = 2; Malawi: n = 3).

Switch-Task

The switch-task assessed the infant's ability to anticipate where a target would appear and the infant's ability to inhibit an initial rule (pre-switch) in favor of a new rule (post-switch phase) (modeled on Kovács and Mehler 2009). After the participant fixated on an attention grabber (a cartoon image of a pink pig face, 5° visual angle), each trial started following a short delay. At the start of each trial two blank rectangles were presented to the left and right together with auditory stimulus for 1000 ms. When the infants made correct gaze anticipations to the location where the target would appear (or after 1000 ms had passed) an audiovisual reward appeared for 2000 ms. The reward appeared on one side (left or right) on the first eight trials and

then on the other side on the last eight trials. Two blocks (i.e., eye tracking session 1 and 2) of 16 trials were presented during the assessment (a total of 16 pre-switch and 16 post-switch trials). A trial was considered valid if it met the pre-processing criteria for the analysis period (i.e., from 0-1000 ms from the onset of the anticipatory period; see Processing of the eye tracking data). Of the valid trials we calculated proportion of correct anticipatory looks. Data were excluded from infants who failed to provide at least 3 valid trials in both the pre- and post-switch phase (Finland: n = 0; Malawi: n = 2).

Attention Disengagement Task

The attention disengagement task assessed the ability of the infant to disengage from a centrally presented stimulus, an emotional face or a control stimulus, to a lateral stimulus using an overlap design, i.e., the lateral stimulus was presented after the central stimulus while the latter stayed on the screen (e.g., Peltola, et al., 2013; Forssman et al., 2014). The face stimuli consisted of color images of two different female faces presenting a happy, or fearful facial expression. The control stimuli was created by randomizing the phase spectrum (i.e., the pixels' positions) of all the face images, controlling for low level visual features (e.g., brightness and amplitude spectrum), thus creating four different control stimuli that corresponded to each face image. For the Finnish sample we used two "typically" Finnish-looking face models and for the Malawian sample we used two "typically" Malawian-looking face models. The validity of the Finnish face stimuli (two models of happy and fearful facial expressions) in terms of belonging to the intended emotional categories and equality of emotional intensity has been shown to be acceptable (Peltola et al. 2009). For the current study, a group of 18 Malawian adults rated all the face stimuli for happiness

and fearfulness on a scale from 1 (very little) to 7 (very much). Pairwise-comparison of the fearful and happy facial expressions across sites revealed no significant difference in ratings of intensity ($p > .05$) for Finnish happy ($M = 6.11$, $SD = 1.47$) and Malawian happy ($M = 5.58$, $SD = 1.26$) expressions, or Finnish fearful ($M = 6.42$, $SD = .96$) and Malawian fearful ($M = 6.31$, $SD = 1.07$) expressions.

Before the start of each trial, the participants were presented with a gaze-contingent attention grabber (a yellow cartoon flower; 5° visual angle). Once the participant fixated on the attention grabber, the trial commenced following a short delay. In each trial the infant was first presented with the central stimuli (subtending 16° and 14° visual angle). After 1000 ms, the central stimulus was flanked by a gaze-contingent lateral stimulus (15° visual angle off-center), an audiovisual reward, on the left or right. The lateral stimulus consisted of a colorful animated movie on a white background embedded between two black squares. When the infant looked at the lateral stimulus, or if 3000 ms had passed from the onset of the lateral stimulus, the movie played for 2000 ms.

The trials were presented in a pseudo-randomized order in which neither the condition (fearful, happy, or control) nor the same target side (left or right) was repeated more than three times in a row. The facial expressions and the control stimuli from one model were presented in the first eight trials in each session, after which the stimulus from the second model was used. The order of the two models was counterbalanced across participants. The participants were presented with two blocks of 16 trials (32 trials in total: 16 control condition trials, 8 fearful condition trials, and 8 happy condition trials).

Similar to our previous studies (e.g., Leppänen et al., 2014), a trial was considered invalid if the infant did not look at the central stimulus for at least 70

percent of the time prior to saccade, if the infant made an anticipatory eye movement (i.e., eye movement commenced < 150 ms after the onset of the lateral stimulus), or an eye movement toward an incorrect location (i.e., the extremely rare cases when the participant's gaze did not move toward the lateral stimulus, which could be caused by a technical artifact). Of the valid trials (see Processing of the eye tracking data for pre-processing criteria), we calculated the proportion of correct saccades from the centrally presented emotional faces and control stimuli (i.e., eye movement toward the lateral stimulus during a time window from 150 to 1000 ms after the onset of the lateral stimulus). This measure gives an index of the infants' attention to emotional cues by examining whether they show the age typical preference of increase attention to emotional faces over the control stimuli, as demonstrated by a decrease in saccades in the emotional face vs. control condition. Data were excluded from infants who failed to provide at least 3 valid trials/condition (Finland: n = 3; Malawi: n = 4).

Processing Speed

As a measure of the infants' processing speed we used a combined reaction time (RT) score averaged across the three eye tracking tasks. RTs were taken from the control condition in the attention disengagement task, from correct anticipatory looks in the switch-task, and from the one-object trials in the visual search task. These tasks/conditions were selected because we wanted to create a more "pure" measure of RT (i.e., the emotional conditions from the attention disengagement task and the distractor conditions from the visual search task were not included because these conditions include potential confounding factors). For an individual task score to be included in the combined RT measure the participant had to meet the eye tracking inclusion criteria for that particular task, as failure to do so indicates that there was

problem with the overall data quality for the task, e.g., because of inattentiveness or excessive head movements. Further, the participant had to contribute valid RT data from at least two of the eye tracking tasks and one participant (Malawian infant) failed to meet this criterion and was therefore not included in this analysis (86 percent of the participants had valid RT data from all three eye tracking tasks; Finland = 90%, Malawi = 81%).

Processing of the Eye Tracking Data

Data reduction, pre-processing and analysis of the eye tracking data were conducted offline by using the *gazeAnalysisLib*, a library of MATLAB (Mathworks, Natick, MA) routines for automated offline analysis of raw gaze data (Leppänen et al., 2014), and custom written MATLAB scripts for each specific eye tracking task. The MATLAB routines are open source and can be downloaded from http://www.uta.fi/med/icl/methods.html. In brief, first a pre-processing of the raw data was conducted. This involved down-sampling the data by applying a 9-sample moving median filter to remove abrupt spikes in the gaze data that were attributable to technical artifacts. The pre-processing also included interpolating segments of missing data with an upper limit of 200 ms (i.e., trials with segments of missing data > 200 ms were rejected). The actual analyses of dependent variables (e.g., processing speed) were further subjected to a number of predetermined and task specific post-verification checks. A trial was excluded from the analysis if: 1) the upper limit of interpolation was violated; and/or 2) the minimum required fixation time for one area of interest (AOI; the participants' gaze is analyzed relative to user-defined areas of interests within the stimulus presentation screen, e.g., area that covers the face stimuli in the attention disengagement task) prior to a saccade was not met; and/or 3) a border

violation took place during interpolation (i.e., data were missing for segments where the gaze shifted between two AOIs interpolation was not performed and the trial was excluded. See Leppänen et al. (2014) for a more detailed description and rationale for the steps involved in the processing of the eye tracking data.

Eye Tracking – result description

Overall, infants at both sites showed a generally expected performance pattern on the three eye tracking tasks (Fig S2 and 3). In the visual search task both the Finnish and Malawian infants had more missed searches (less correct responses) in the conjunction condition with two types of distractors compared to the condition with one type of distractor (distractor condition), and few failed searches in the condition without distractors (one-object condition) For the switch-task, both groups showed increased learning over time in the pre- and post-switch phase and also the expected perseveration (i.e., initial poorer performance) on the post-switch trials. Finally, in the attention disengagement task both Finnish and Malawian infants had a higher proportion of missing saccades (less correct responses) in the emotion conditions compared to in the control condition.

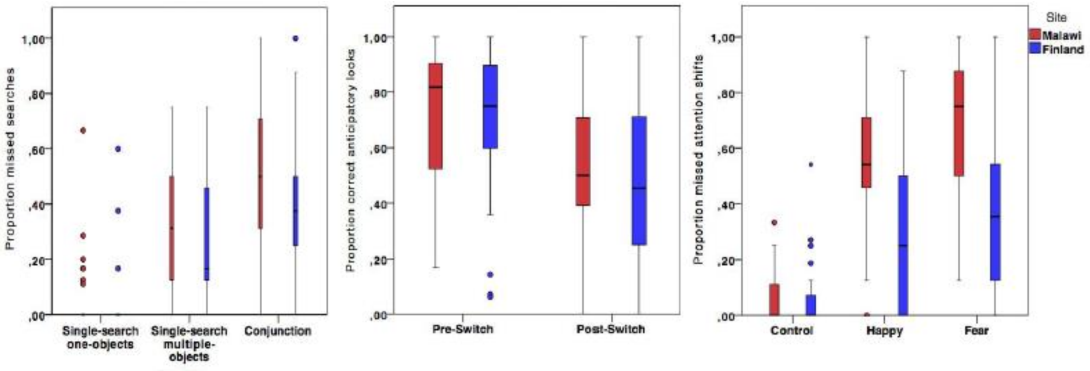

Figure S1. Proportion of correct responses in the eyetracking tasks.

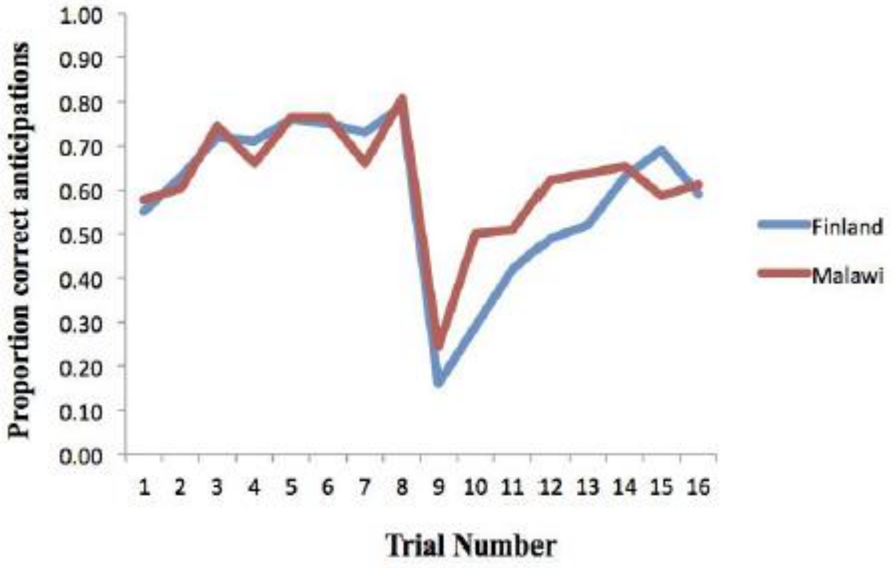

Figure S2. Proportion of correct responses in the switch-task across individual test trials.

Structured Observation of Social Communication Skills

The infants' ability to initiate and respond to social communication cues was assessed with a structured observation method and based on two tasks, *Alternating Gaze* and *Gaze Following*, from the Early Social Communication Scale (ESCS; Mundy et al. 2003). During this assessment, the child was seated on a marked spot on the floor in front of his/her mother, facing the experiment leader. In the *Alternating Gaze* task the experiment leader presented a moving toy (e.g., a shaking rattle) on the floor in front of the infant from a 105 cm distance for 6 s. After 6 s had elapsed the infant was then allowed to manipulate the toy briefly. A total of three toys were presented three times in a row (at different times) during the assessment (total number of trials = 9). As a measure of the ability to initiate social communication, we calculated the number of alternating gazes between the activated toy and the experiment leader's line of gaze during a 6 s time period from the start of the activation of the toy. In the *Gaze Following* task the experiment leader looked and

pointed for 6 seconds in turn to four objects (located 90 and 45 visual degrees to the left and right of the infant). Two sets of four trials were presented during the assessment (total number of trials = 8). As a measure of the ability to respond to social communication we calculated the proportion of trials in which the infants looked to the correct object.

The assessment was recorded with two video cameras and coded off-line. Inter-rater reliability between two coders was calculated from 22% of the sample (randomly selected) and was .98 (*rho*) and .73 (*rho*) for proportion of alternating gaze and proportion of gaze following, respectively. Based on the video records, four participants (Malawian) were excluded from the analysis of the structured observation assessment because of technical error (n=1) or error in the administration of the tasks (n=3).
